# Epithelioid Hemangioendothelioma Presenting as Necrotizing Pneumonia

**DOI:** 10.7759/cureus.39328

**Published:** 2023-05-22

**Authors:** Thao Nguyen, Fatima Chagani, Majd Khasawneh, Tawfiq Khasawneh, Faread Jamalifard

**Affiliations:** 1 Internal Medicine, University of Florida College of Medicine, Gainesville, USA; 2 Pulmonology and Critical Care, University of Florida College of Medicine, Gainesville, USA; 3 Internal Medicine, University of Jordan, Amman, JOR

**Keywords:** pulmonary embolus, rare cancer, lung cancer, epithelioid hemangioendothelioma, necrotizing pneumonia

## Abstract

Epithelioid hemangioendothelioma (EHE) is a very rare vascular neoplasm that is often asymptomatic. A 40-year-old woman presented to the emergency department for evaluation of a nonproductive cough, chest pain, and dyspnea. A chest computed tomography angiography (CTA) demonstrated necrotizing pneumonia, a loculated left-sided pleural effusion, and an acute pulmonary embolus. She was started on broad-spectrum intravenous antibiotics and heparin infusions, and a chest tube was placed. After minimal improvement in her pleural effusion following instillation of fibrinolytics, she underwent video-assisted thoracoscopic surgery with decortication, and a pleural biopsy was performed. Her presenting symptoms resolved shortly thereafter. Following discharge, surgical pathology resulted in a diagnosis of EHE. She was not a candidate for surgical resection and remained under surveillance. A year later, she was found to have metastatic disease, and radiotherapy was initiated. Our case, which presented as necrotizing pneumonia associated with pulmonary EHE, highlights the challenges in diagnosing this disease given its extreme rarity and discusses its management.

## Introduction

Epithelioid hemangioendothelioma (EHE) is a vascular neoplasm composed of epithelioid or histiocytoid cells with endothelial characteristics [1]. It is a rare neoplasm with a reported prevalence of one in one million. Roughly 30% of cases involve the lungs, and these have a wide range of radiographic findings [1-3]. A definitive diagnosis is made by histopathologic examination and immunohistochemistry staining of the involved tissue [4-6]. Given its low prevalence and nonspecific symptomatology, it is rarely considered in the differential diagnosis of lung consolidation. Our case not only highlights the challenges in EHE’s diagnosis and management but also exemplifies the classic epidemiologic features associated with it.

## Case presentation

The patient was a 40-year-old woman with a past medical history of scoliosis resulting in chronic back pain who presented to the emergency department for evaluation of progressive dyspnea, a nonproductive cough, and left-sided chest pain for five days. She denied any weight loss, night sweats, fatigue, or chills. She reported an eight-pack-year smoking history prior to quitting seven years ago. She denied any recent travel or known environmental or occupational exposures. Her family history was non-contributory. Her chronic medications included cyclobenzaprine, ibuprofen, and paroxetine. Notably, she had experienced an acute worsening of her chronic back pain two months prior. Despite denying any pulmonary symptoms at that time, she was diagnosed with pneumonia after a chest x-ray demonstrated a left apical opacity. She was treated with oral doxycycline for seven days, which resolved her acute back pain.

In the emergency department, she was ill-appearing, febrile to 38.8°C, and tachycardic to 146 beats per minute. Her respiratory rate was 27 breaths per minute, and her oxygen saturation was 98% in room air. Respiratory examination revealed decreased anterior and posterior breath sounds over the left lung field diffusely with inspiratory crackles and no accessory muscle use. She had no rashes, joint swelling, clubbing, or lower extremity edema.

Initial labs revealed a white blood cell count of 13.6 K/uL with 82.8% neutrophils. Lactic acid and procalcitonin were normal. High-sensitivity C-reactive protein was markedly elevated at 453 mg/L, and the erythrocyte sedimentation rate was 75 mm/hr.

**Figure 1:**
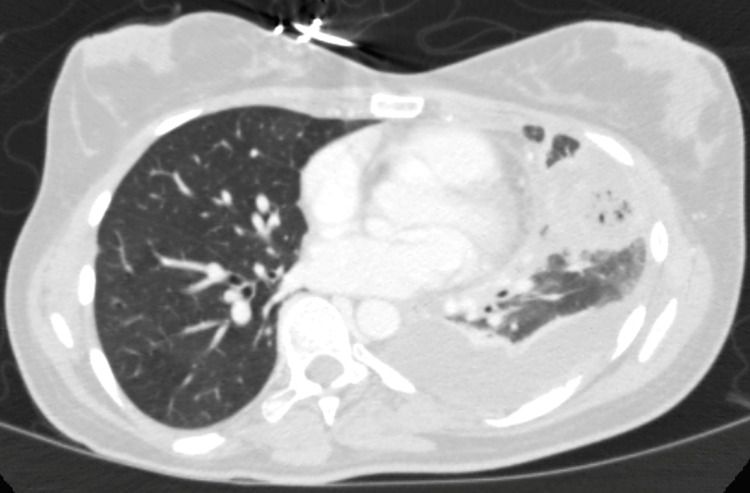
CTA chest (lung window) demonstrating left lower lobe necrotizing pneumonia with moderate-sized loculated left pleural effusion and abscess CTA: computed tomography angiography

Initial imaging investigations included a chest x-ray that revealed left lower lobe consolidation. Subsequently, a chest CT angiogram pulmonary embolism protocol demonstrated a left lower lobe necrotizing pneumonia with early abscess formation and possible extension into the mediastinum, a loculated left-sided pleural effusion, and a left upper lobe segmental artery pulmonary embolus (Figure 1).

Further workup included antinuclear antibodies (ANA) IgG and HIV antigen tests, both of which were negative. Her respiratory pathogen polymerase chain reaction (PCR) panel, Legionella urinary antigen, and aerobic and anaerobic blood cultures were all negative.

The patient was evaluated by pulmonology and had a chest tube placed with drainage of serosanguinous fluid that was consistent with an exudative effusion based on all three of Light’s criteria. Pleural fluid analysis was notable for normal pH, normal glucose, and a white blood cell differential of 75% lymphocytes and 25% polymorphonuclear cells. Aerobic, anaerobic, and acid-fast bacilli (AFB) cultures had no growth, and cytology was negative for malignancy. Her left-sided pleural effusion minimally improved following the instillation of intrapleural tissue plasminogen activator and dornase alfa daily for three consecutive days; thus, thoracic surgery was consulted. The patient underwent left video-assisted thoracoscopic surgery (VATS) decortication, during which the pleural surface was noted to be abnormal with patchy round deposits that were biopsied.

**Figure 2:**
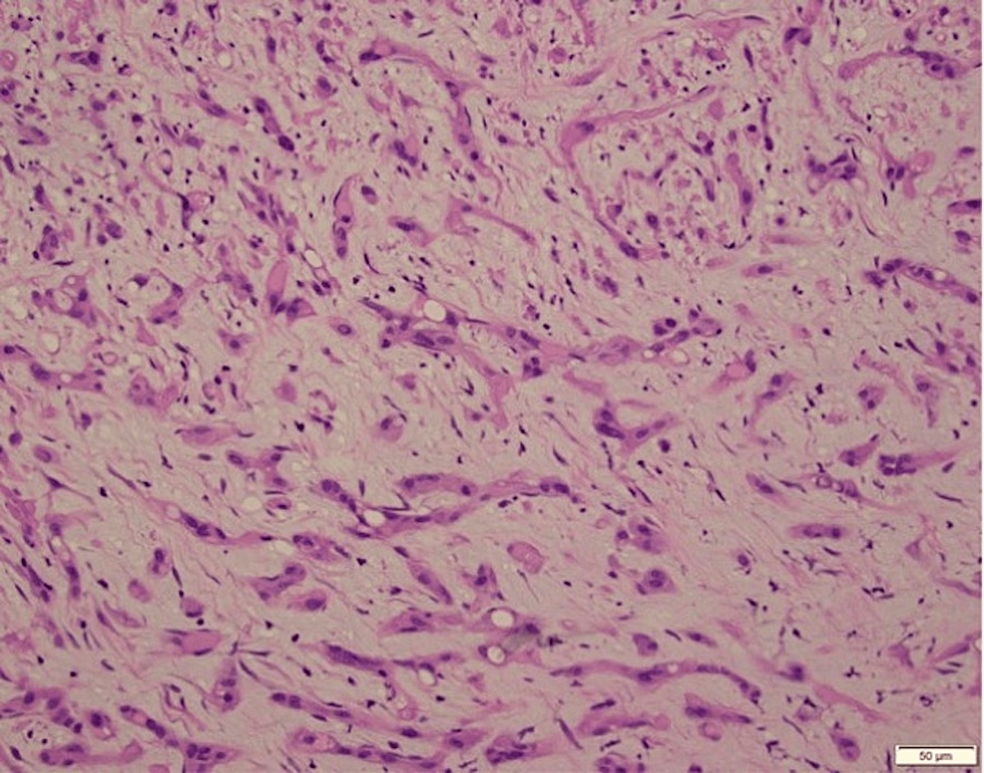
Hematoxylin and eosin staining demonstrates epithelioid cells mostly in cords deposited in a hyalinized and myxoid stroma. Scattered cells show cytoplasmic vacuoles known as "blister cells".

**Figure 3:**
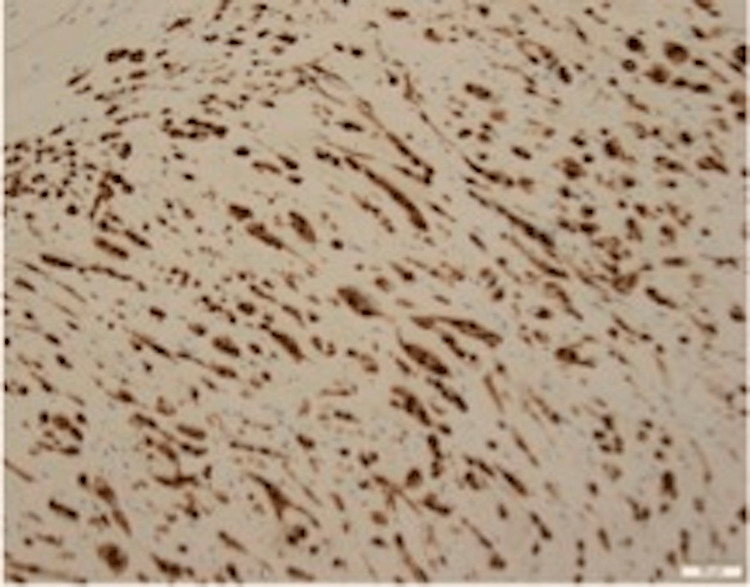
Immunohistochemical stain for CAMTA-1 is positive in neoplastic cells.

Following management of her pleural effusion, anticoagulation for her pulmonary embolus, and empiric antibiotics, the patient’s presenting symptoms resolved, and she returned to her baseline. Following discharge, her pleural biopsy histopathologic examination yielded tumor cells demonstrating positive immunohistochemistry staining of CD 31 and CAMTA-1 (calmodulin binding transcription activator 1), and targeted RNA sequencing demonstrated WWTR1-CAMTA-1 (WW domain containing transcription regulator 1, calmodulin binding transcription activator 1) fusion (Figures 2-3).

Following review by the thoracic multidisciplinary tumor board, she was deemed not to be a candidate for complete resection given the patchy and scattered pattern of the lesions. Since she became asymptomatic following management of her necrotizing pneumonia, pleural effusion, and pulmonary embolus and without established guidelines for the treatment of EHE, a shared decision was made with her oncologist to observe the patient with serial imaging without treatment.

The patient remained asymptomatic with follow-up at three-month intervals with repeat chest CT scans. These showed stable disease without progression for one year. After one year, her pain worsened, and subsequent images revealed new liver and pancreatic findings concerning metastatic disease. She was started on radiation therapy, followed by palliative care for assistance with symptom management, including pain control.

## Discussion

EHE is a low- to intermediate-grade angiosarcoma originating from vascular endothelial cells and characterized by the pathognomonic fusion of the WWTR1 and CAMTA1 genes [3, 7]. The median age of onset is 36 years, and it mostly affects women. One-third of cases primarily involve the lungs [3]. Pulmonary EHE is most often asymptomatic and incidentally found on imaging; however, in symptomatic patients, cough, dyspnea, pleuritic chest pain, and hemoptysis are common presentations [1, 3]. Pulmonary EHE’s radiographic findings include multiple unilateral or bilateral perivascular nodules or opacities, lymph node involvement, interlobular septal thickening, pleural effusions, ground-glass attenuation, and calcifications [1, 2]. Given the low prevalence and lack of pathognomonic symptoms or radiographic findings, it is rarely considered in the differential for lung pathologies. Diagnosis requires histopathologic exam and immunohistochemistry staining for WWTR1 and CAMTA1 of tissue obtained by biopsy or surgical resection, which should also be stained for important vascular and endothelial markers including factor VIII-related antigen, CD31, CD34, and Fli-1 [1, 3-6].

The overall mean survival for patients with EHE is 4.6 years; asymptomatic pulmonary EHE has a median survival of fifteen years [1]. A systematic review of pulmonary EHE demonstrated a worse prognosis with ages above 55 years, male sex, symptomatic disease, multi-organ involvement, and the presence of pleural effusion with pleural involvement at the time of diagnosis [4].

The European Society of Medical Oncology recommended surgical resection for limited disease due to potential spontaneous regression [1,3,8,9]. While the optimal length and frequency of follow-up remain unknown, biannual whole-body CT is recommended for five years after diagnosis, followed by yearly scans if the disease remains stable [8]. Metastatic and symptomatic diseases can be treated with chemotherapy, immunotherapy, anti-angiogenic therapy, or radiation therapy [1,3,8]. The role of chemotherapy in advanced or metastatic disease is unclear; some patients demonstrate complete responses, while in other cases, chemotherapy has been associated with poor outcomes [10]. Anti-angiogenic therapy such as pazopanib has demonstrated some long-term disease control, but only in two anecdotal cases [11]. Vascular endothelial growth factor receptor (VEGFR) inhibitors such as bevacizumab and sorafenib have been effective and well tolerated in a phase II trial of metastatic advanced EHE [12]. Lastly, radiation therapy tends to be favorable as it is well tolerated and can offer local pain control, improving the patient’s quality of life [1].

In our case, the patient presented with isolated lung involvement that was considered unresectable given the patchy and scattered pattern of the lesions. Her initial symptomatology resolved following treatment of associated acute processes, including pneumonia, pulmonary embolism, and management of pleural effusion. She remained asymptomatic on follow-up with her oncologist, where an initial shared decision was made to follow her closely with serial imaging after treatment. Unfortunately, a year later, the patient had evidence of metastatic disease in her liver and pancreas. Following a discussion with her oncologist, she was offered radiation therapy or systemic bevacizumab. She chose radiation therapy in hopes of a potentially greater benefit in pain control, as this was her most burdensome symptom. She is also being followed by palliative care for assistance with symptom management, including pain control.

This case questions past literature that supports surveillance alone for limited diseases [9]. The presence of pleural effusion at initial presentation, which is associated with a worse prognosis, made surveillance alone a higher risk [4]. It is unknown if her outcome would have been different if she had been started on systemic therapy or radiation therapy following her initial diagnosis. Therefore, future research is needed to help guide the treatment of EHE, especially in unresectable, asymptomatic patients.

Upon reviewing the literature, only two other cases of pulmonary EHE initially presented with pneumonia, both pediatric cases. In the first case, the patient had pneumonia, with further workup leading to the diagnosis of pulmonary EHE. Unfortunately, this patient died six months after diagnosis, despite resection and chemotherapy [13]. In the second report, a teenager presented with necrotizing pneumonia and was found to have EHE [14]. Our case similarly demonstrated radiographic evidence of necrotizing pneumonia; however, subsequent pathology, microbiology, and pleural fluid analysis did not support this imaging finding. As such, it is the authors’ opinion that the patient’s fever on presentation was not related to an infectious etiology but instead was multifactorial due to her pulmonary embolus, inflammatory response to necrosis, and underlying malignancy. It is also the authors’ opinion that, given her pulmonary embolism’s location, it is unlikely to be a direct consequence of the vascular neoplasm originating from the correlating endothelium but instead due to her hypercoagulable state from malignancy.

## Conclusions

Pulmonary EHE is most often asymptomatic and has a wide range of non-specific radiographic findings, including multiple perivascular nodules, lung masses, and pleural effusions. As a result, it should be considered in the differential diagnosis when these findings are unexplainable, especially in women in their third and fourth decades of life. Localized pulmonary EHE is best managed with complete surgical resection. Frequent follow-up is recommended for asymptomatic patients with unresectable disease without poor prognostic factors. For those who are symptomatic or who demonstrate progressive disease, systemic chemotherapy, immunotherapy, radiation therapy, and/or anti-angiogenic therapy are recommended.
